# Research Review: Do antibullying interventions reduce internalizing symptoms? A systematic review, meta‐analysis, and meta‐regression exploring intervention components, moderators, and mechanisms

**DOI:** 10.1111/jcpp.13620

**Published:** 2022-04-26

**Authors:** Carolina Guzman‐Holst, Mirela Zaneva, Chloe Chessell, Cathy Creswell, Lucy Bowes

**Affiliations:** ^1^ 6396 Department of Experimental Psychology University of Oxford Oxford UK; ^2^ 6816 School of Psychology and Clinical Language Sciences University of Reading Reading UK; ^3^ 6396 Department of Psychiatry University of Oxford Oxford UK

**Keywords:** School intervention, antibullying, internalizing, depression, anxiety, children, adolescents

## Abstract

**Background:**

Effective antibullying interventions may reduce the impact of bullying on young people’s mental health. Nevertheless, little is known about their effectiveness in reducing internalizing symptoms such as anxiety or depression, and what factors may influence intervention effects. The aim of this systematic review, meta‐analysis, and metaregression is to assess the effects of school‐based antibullying interventions on children’s and adolescent’s internalizing symptoms. The secondary aims are to explore potential moderators, intervention components, and reductions in bullying as mediators of intervention effects on internalizing symptoms.

**Methods:**

We searched nine databases: PsycINFO, Web of Science, ERIC, SCOPUS, CINAHL, Medline, Embase, ProQuest, and Cochrane Library, and performed an author search of included studies in English from January 1983 to April 2021. We included studies that evaluated school‐based antibullying interventions using controlled designs and reporting on both bullying and internalizing outcomes. Random‐effects and metaregression models were used to derive Hedges g values with pooled 95% CIs as estimates of effect size and to test associations between moderator variables and effect size estimates. Path analysis was used to test potential mediation using effect size measures of victimization, perpetration, and internalizing outcomes. Quality and risk of bias were assessed using Cochrane collaboration tools.

**Results:**

This review included 22 studies with 58,091 participants in the meta‐analysis. Antibullying interventions had a very small effect in reducing overall internalizing symptoms (ES, 0.06; 95% CI, 0.0284 to 0.1005), anxiety (ES, 0.08; 95% CI, 0.011 to 0.158), and depression (ES, 0.06; 95% CI, 0.014 to 0.107) at postintervention. The reduction in internalizing symptoms did not vary significantly across geographic location, grade level, program duration, and intensity. The intervention component ‘working with peers’ was associated with a significant reduction, and ‘using CBT techniques’ was associated with a significant increase in internalizing outcomes. Bullying victimization and perpetration did not mediate the relationship between intervention condition and internalizing outcomes.

**Conclusions:**

Antibullying interventions have a small impact on reducing internalizing symptoms. Ongoing development of antibullying interventions should address how best to maximize their impact on internalizing symptoms to safeguard young people from the damaging mental health outcomes of bullying.

## Introduction

Depression and anxiety (internalizing) disorders are among the most frequently diagnosed psychiatric conditions in childhood and are the leading causes of disability and burden in young people (Erskine et al., 2015; Ghandour et al., 2019). Longitudinal studies have shown continuity in internalizing problems from childhood to adulthood (Hofstra, Van der Ende, & Verhulst, 2000) and higher lifetime prevalence rates compared to any other psychiatric disorder (Kessler et al., 2005) suggesting the need to identify modifiable risk factors that can be targeted in interventions during early life. Bullying victimization appears to be one of the most tractable risk factors for internalizing symptoms. Specifically, research has shown that over a quarter of cases of depression at age 18 might be attributable to bullying victimization during early adolescence (Bowes, Joinson, Wolke, & Lewis, 2015). Studies suggest that young people with internalizing difficulties are more likely to be bullied and young people who are bullied are more likely to develop internalizing difficulties, even after adjusting for baseline internalizing difficulties (Arseneault et al., 2008; Singham et al., 2017; Stapinski, Araya, Heron, Montgomery, & Stallard, 2015). If this relationship is causal as it has been suggested, antibullying interventions offer an important way to safeguard young people’s mental health.

In relation to its mental health impact, school‐based bullying is recognized as a major public health issue. A study by UNESCO (2019) found that 32% of children globally had experienced bullying victimization on one or more days over the past month and 7.3% of young people had experienced bullying on 6 or more days over the same time period (UNESCO, 2019). It is important to highlight that while most children experience low and stable trajectories of peer victimization throughout the school years, other children experience chronic and increasing levels of peer victimization (Barker et al., 2008; Biggs et al., 2010). Indeed, research has shown that this latter group is also the most at risk of prior and subsequent internalizing problems (Sukhawathanakul & Leadbeater, 2020).

A person characterizes bullying repeatedly being exposed to aggressive or negative actions by another person intending to cause physical harm, psychological distress, or humiliation (Olweus, 1993). Different types of bullying include traditional forms such as physical, relational, emotional, and verbal bullying, while other nontraditional forms such as cyber bullying can take place at any time and context. In childhood, traditional bullying victimization and perpetration predominantly occurs within the school context (Department for Education, 2018) and have been associated with higher mean prevalence rates (35%) than nontraditional bullying victimization and perpetration (15%) across 80 studies (Modecki, Minchin, Harbaugh, Guerra, & Runions, 2014). This highlights the important role of schools as grounds for antibullying prevention.

### Effectiveness of school antibullying interventions

Many school‐based interventions have been devised and implemented to reduce school bullying. Gaffney, Farrington, and Ttofi’s (2019) meta‐analysis showed that, on average and when compared with school usual practice, these programs decreased bullying by 19%–20% and victimization by 15%–16% (Gaffney et al., 2019). Yet, despite evidence showing an association between bullying victimization and internalizing symptoms (Reijntjes, Kamphuis, Prinzie, & Telch, 2010), not all interventions measure or report their intervention’s effects on mental health. For those who do report them, findings have been mixed with some suggesting that interventions can reduce psychological distress while others find no such effects (Bonell et al., 2018; Dempsey, 2009; Hoglund, Hosan, & Leadbeater, 2012; Williford et al., 2012). Recently, Fraguas et al. (2021) conducted a systematic review and meta‐analysis assessing the effect of school antibullying interventions on mental health and found an overall effect size using Cohen’s *d* of −.205 (95% CI, −0.277 to −0.133). However, they only examined randomized controlled trials (RCTs) in published journal articles and used a broad definition of mental health including proxies such as mental health symptoms, quality of life, self‐esteem, self‐blame, and social skills. It is likely that such broad proxies for mental health resulted in larger effect sizes and more heterogeneity, but less specificity in identifying intervention effects on internalizing difficulties. Furthermore, they did not look at intervention components or whether reductions in victimization mediated mental health outcomes. This information could aid in determining what specific components improve intervention effects on internalizing and through what mechanisms they potentially operate. A systematic review and meta‐analysis to quantify intervention effects on internalizing symptoms is warranted.

### Moderating and mediating effects of antibullying interventions

Very little is known about the moderators and mechanisms that play a role in determining intervention effects on internalizing symptoms. Several studies have shown that type of intervention such as whole school versus targeted (Gaffney et al., 2019), age (Fraguas et al., 2021; Yeager, Fong, Lee, & Espelage, 2015), informant type (Shakoor et al., 2012), location (Gaffney et al., 2019), and number of components (Gaffney, Ttofi, & Farrington, 2021a, 2021b) may determine the effectiveness of interventions at reducing bullying. This has not been examined yet in the context of internalizing symptoms and could provide important information about what type of antibullying interventions can improve mental health.

Understanding which specific intervention components are more effective at reducing children’s internalizing symptoms can also provide important recommendations on how to maximize the effectiveness of future antibullying programs. In a comprehensive Campbell review, Farrington and Ttofi (2009) found that increased teacher training, increased duration and intensity of the intervention, parental involvement, playground supervision, and using disciplinary methods were associated with a decrease in both bullying victimization and perpetration. Working with peers was associated with an increase in victimization, although this was recently found to not be the case in a follow‐up review (Gaffney et al., 2021a, 2021b). This follow‐up review found that interventions with a whole‐school approach, antibullying policy, classroom rules, information for parents, informal peer involvement, work with victims, cooperative group work, and mental health approaches were associated with larger reductions in bullying perpetration, and informal peer involvement and information for parents were associated with larger reductions in bullying victimization. The *absence* of socioemotional skills components was associated with significantly larger reductions in both perpetration and victimization outcomes. Program richness (i.e. the number of intervention components used) was not significantly associated with changes in perpetration or victimization (Gaffney et al., 2021a, 2021b). It is unclear whether these components would have similar effects on internalizing outcomes.

In terms of mediating effects, a factor that may explain the difference in intervention’s effects on internalizing symptoms is the extent to which the interventions change the prevalence of bullying perpetration and victimization (Palladino, Nocentini, & Menesini, 2019). That is, interventions that achieve greater reductions in bullying victimization should theoretically lead to greater reductions in internalizing symptoms. These mechanisms have not yet been examined across multiple antibullying programs, yet they could inform researchers and public health professionals of how these programs work and target mental health moving forward.

### The current study

This review aims to answer the following questions:

1. Are school‐based antibullying interventions associated with improvement or deterioration of internalizing symptoms? Do effect sizes vary according to type of internalizing symptom (anxiety or depression), type of intervention (targeted vs. whole school), age group (children or adolescents), and study quality (low vs. high risk of bias)?
2. Do specific study and participant characteristics such as duration and intensity of intervention, type of informant for internalizing measures, school grade level, location, and number of intervention components moderate the effect of antibullying interventions on internalizing outcomes?
3. Do bullying victimization and perpetration prevalence outcomes mediate the effect of antibullying interventions on internalizing symptoms?
4. Are specific intervention components independently associated with improving or worsening internalizing outcomes?

## Methods

### Protocol and registration

This systematic review and meta‐analysis was preregistered with PROSPERO (CRD42020191857) and uses PRISMA reporting guidelines. Deviations from our preregistration protocol are detailed in Appendix S1.

### Search strategy

For this review, we searched nine databases: PsycINFO, Web of Science, ERIC, SCOPUS, CINAHL, Medline, Embase, ProQuest, and Cochrane Library from January 1983, as this is when bullying intervention notably emerged (Olweus, 1993), to May 2021. This search was complemented by an author search and a meta‐analysis search to obtain as many evaluations of known intervention programs as possible. To decrease publication bias, we included theses and dissertations in addition to book chapters and journal articles written in English. The specific search strategy is described in Appendix S2.

### Eligibility criteria

We included studies that evaluated an antibullying intervention delivered in schools to children and adolescents aged 4–19 years old. To meet eligibility criteria, the study stated a clear definition of bullying, measured bullying perpetration, or victimization as a primary outcome, and measured internalizing symptoms as either a coprimary or secondary outcome postintervention. Studies reported effect sizes or relevant data that allowed for the computation of effect sizes for bullying and internalizing. Bullying and internalizing outcomes were measured using a validated, standardized, age‐appropriate measure (internalizing scales must have been completed by child, parent, or teacher, and show good internal consistency (α = .6 or above), previous evidence of construct validity, and ±1 year within the suggested age range). The study design included an experimental group that received the intervention with a control group that did not. The experimental and control group could have been in the same school or in different schools. If there was more than one experimental group (e.g. two groups using different interventions) in the same study, there must have been a control group not receiving any intervention. The types of designs included were as follows: randomized experiments, experimental–control comparisons with pre‐ and postmeasures of bullying and internalizing symptoms between groups, and other experimental–control comparisons. For trials with multiple publications, the most relevant study was included after assessing them against all eligibility criteria. For studies that reported on two or more experimental intervention groups or one or more control groups, we followed Cochrane recommended guidelines (Chapter 16.5.4) and either selected the most relevant group/condition or split the shared group into two or more groups with smaller sample sizes to make independent comparisons.

### Study selection

**Figure 1:**
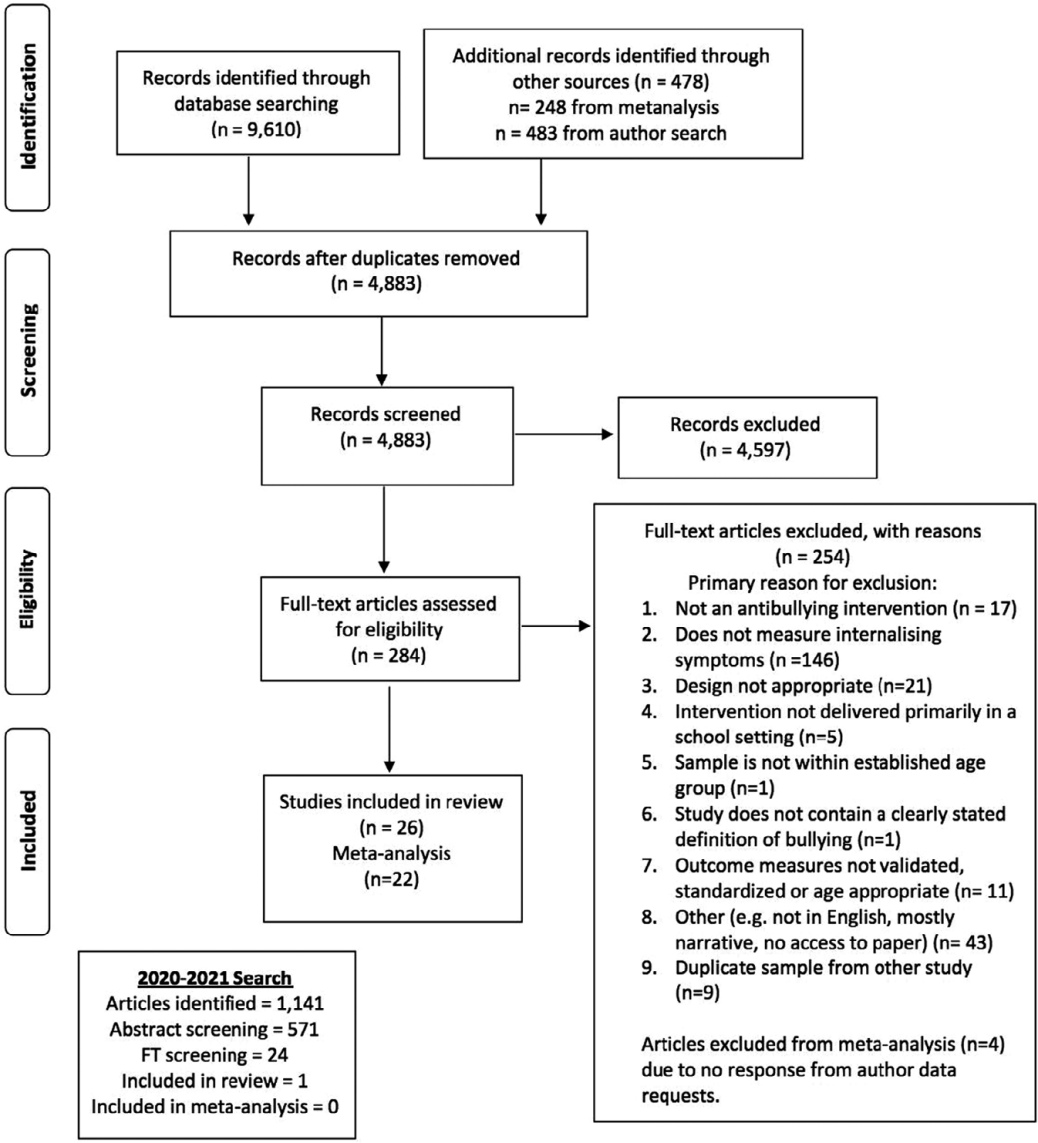
Prisma flow chart

A flow chart of the study selection process is shown in Figure 1. We identified 10,751 records (9,610 from the first search and 1,141 from the 2020–2011 search) from the electronic databases and an additional 478 records from reviews and author searches from the first search. A total of 5,454 records were retained after duplicates were removed following both searches. Two authors (CGH and MZ) used Raayan Software to independently screen titles and abstracts and identify records for full‐text screening. Interrater reliability between the two review authors was calculated and classified as ‘almost perfect agreement’ (*k* = .85; Landis & Koch, 1977). Disagreements were resolved between two authors (CGH and MZ) and when consensus was not reached, a third review author (LB or CCr).

### Data extraction

Two authors (CGH and CCh) independently extracted study characteristics, outcome data, intervention components, and potential moderators from eligible articles. Intervention components were identified based on elements found during article screening in combination with Farrington & Ttofi's (2009) element list. We consulted with the evaluators of the various programs by sending them our coding and description of the elements (see Appendix S3). We received author ratings for 18 of the 22 evaluations included in the meta‐analysis (see Table S1). Missing information for key variables from included studies were also requested from authors.

### Assessment of methodological quality (risk of bias)

This review used the Cochrane collaboration's tools for assessing risk of bias for cluster randomized controlled trials (RoB‐2 cluster randomized), individual cluster randomized controlled trial (RoB‐2), and cross‐over trials (RoB‐2 cross‐over). Two reviewers (CGH and MZ) independently rated the quality of all included studies. Any discrepancies were initially discussed by the two reviewers and a third review author (LB or CCr) when consensus was not reached.

### Effect measures

Hedge’s *g* effect sizes and 95% confidence intervals were considered the primary outcome of the meta‐analysis. They were calculated using post‐intervention sample size, means, and standard deviation, or regression coefficients from each included study. As many studies involved clustered designs, we corrected the sample size for clustering using Cochrane guidelines. When intracluster correlation coefficients (ICC) were not available, we used an average estimate from the literature (ICC = 0.025). For studies reporting multiple internalizing outcomes, effect sizes, and respective variances were averaged to obtain a single‐effect estimate using equations reported elsewhere (López‐López, Page, Lipsey, & Higgins, 2018).

### Data synthesis and statistical analysis

For the meta‐analysis, main results for overall internalizing symptoms were examined using random‐effect models with an inverse‐variance approach and DerSimonian–Laird estimator and subgroups were examined using mixed‐effect models. Subgroup analysis included type of internalizing symptom (anxiety or depression), type of intervention (targeted vs. whole school), age group (children or adolescents), and study quality (low vs. high risk of bias). The *Q* and *I* ^2^ statistics were used to assess heterogeneity between study results. R packages *meta*, *metafor*, and *dmetar* were used.

To assess moderation and intervention component effects, we performed metaregression analysis for each moderator and intervention component determined a priori. For metaregression, it is recommended that each covariate contain at least 10 studies (Borenstein, Hedges, Higgins, & Rothstein, 2011), therefore type of informant was not examined as only 2 studies used a different type of informant that was not self‐reported.

Lastly, to examine the extent bullying victimization and perpetration prevalence outcomes mediate intervention effects on internalizing symptoms, we used mean pre–post scores to calculate Hedges’ *g* effect sizes in bullying and internalizing outcomes for control and intervention groups, for each study, in a school‐level mediation analysis. We calculated the direct effect ‘*c*’ (effect of intervention condition on internalizing), indirect effect ‘a*b’ (effect of intervention on victimization/perpetration and effect of victimization/perpetration on internalizing), and the total effect ‘*c* + (a*b)’. To test the significance of the indirect effect, we used bootstrapping procedures. Unstandardized indirect effects were computed for each of 1,000 bootstrapped samples. Two studies (Bonell et al., 2018, and Williford et al., 2012) were not included in the mediation analysis as they did not have baseline measures of internalizing symptoms. These analyses were conducted using the *lavaan* package in R.

## Results

### Search results

A total of 5,454 abstracts were reviewed. Of these, 27 articles were included for full‐text review and 22 met criteria for the meta‐analysis. The process of study selection is presented in Figure 1.

### Study characteristics

Study characteristics are presented in Table S2. In total, 27 studies conducted between 1999 and 2020 were included in the review. Studies ranged in sample size from 24 students in targeted interventions to 7,741 students in whole‐school interventions. Forty‐eight percent of studies targeted grades 1–6, and 52% of studies targeted grades 7–12. Participants’ mean age was 10.5 years. Fifty‐nine percent of studies had interventions delivered by school staff or teachers and all were based in schools. Seventy percent of studies included whole‐school interventions, 26% targeted interventions, and 3.7% included both whole‐school and targeted components. Most studies came from high‐income countries, Australia (*n* = 7), United States (*n* = 5), England (*n* = 4), Canada (*n* = 3), Netherlands (*n* = 2), Finland (*n* = 1), Italy (*n* = 2), and Germany (*n* = 1), with one study from a middle‐income country (China), and one from a lower‐middle‐income country (Pakistan). Only one study (INCLUSIVE trial, Bonell et al., 2018) provided program costs and two others (KiVA, Huitsing et al., 2019; Williford et al., 2012) provided costs from the main trials of the study. Costs for these trials were estimated to be less than £100 per student per year, without including annual costs and once only costs (Huitsing et al., 2019; Persson, Wennberg, Beckman, Salmivalli, & Svensson, 2018).

A summary of study design characteristics is shown in Table S3. Studies varied in terms of study design characteristics, 51.9% (*n* = 14) of studies were cluster randomized, 11.1% (*n* = 3) were individually randomized, 29.6% (*n* = 8) were not randomized and 7.4% (*n* = 2) were cross‐over trials. Interventions included in these studies varied considerably in their measure of internalizing symptoms. 44.4% (*n* = 12) of studies had a specific measure of anxiety, 55.5% (*n* = 15) studies had a specific measure of depression and 44.4% (*n* = 12) had a general measure of internalizing symptoms (e.g. SDQ emotional problems subscale). More than half of the studies had more than one measure of internalizing symptoms.

### Quality assessment and risk of bias in included studies

The quality of included studies varied considerably. Ten studies were rated as having a low risk of bias, 6 were rated as having some concerns for risk of bias, and 11 studies were rated as having a high risk of bias. For studies included in the meta‐analysis, eight were rated as having a low risk of bias, four were rated as having some concerns, and seven studies were rated as having a high risk of bias. A detailed quality assessment is shown in Figure S1.

### Meta‐analysis and subgroup analysis

**Figure 2:**
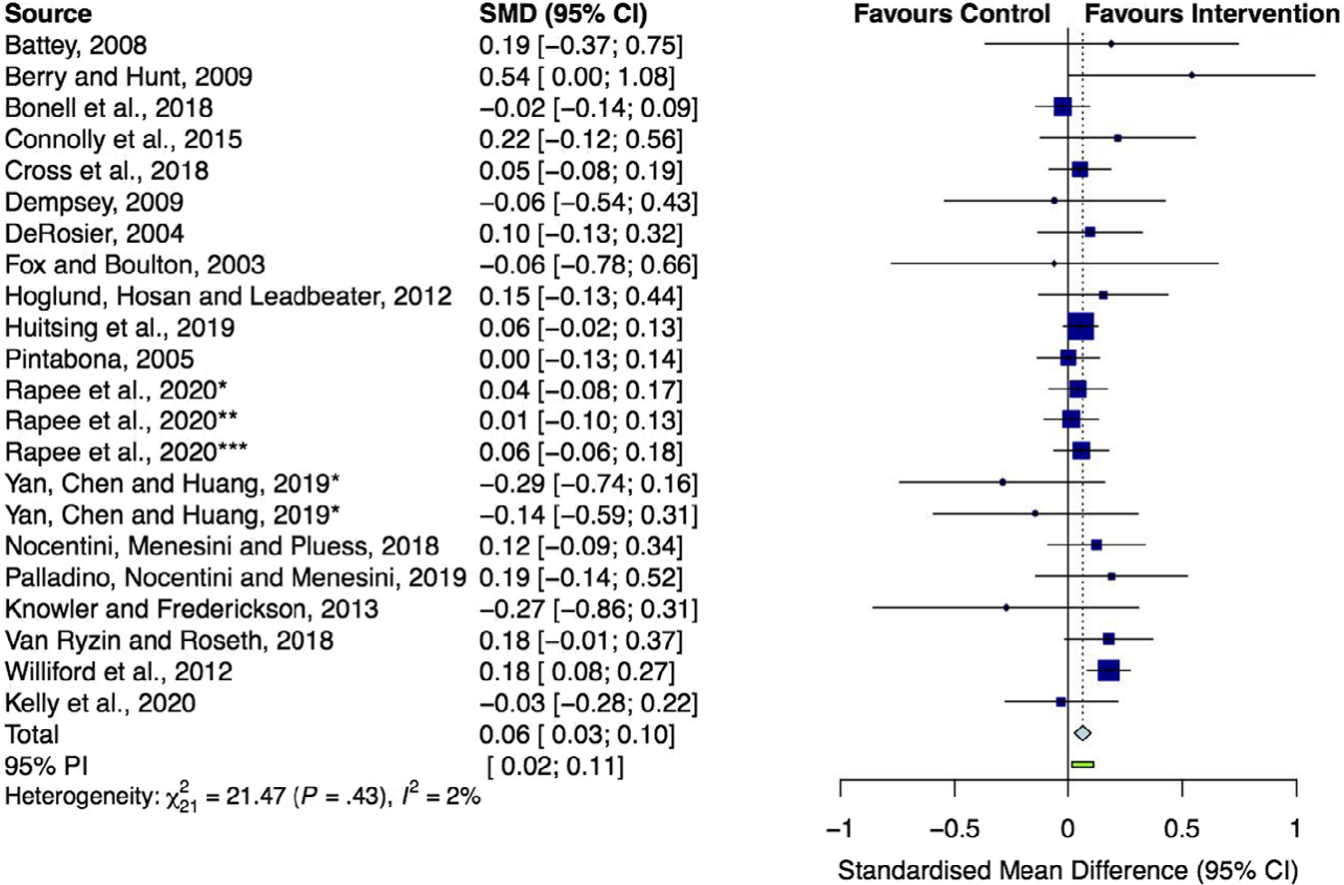
Impact of school‐based antibullying interventions on overall internalizing symptoms

Fifteen of the 22 effects (68%) were larger than 0, indicating a reduction in overall internalizing symptoms favoring the intervention. The overall mean effect on internalizing symptoms was 0.06 (95% CI, 0.0284 to 0.1005; *z* = 3.50; *p* < .001), indicating that antibullying interventions had very little effect on overall internalizing symptoms. Our two heterogeneity measures indicated that there was low heterogeneity in our data (*Q* = 21.47, *df* = 21, *p* = .4304; *I* ^2^ = 2.2%, 95% CI: 0% to 47.4%). Figure 2 shows the forest plot for the overall and individual effect sizes.

### Subgroup analysis

Subgroup analysis for specific internalizing symptoms included 12 studies with measures of depression and 14 studies with measures of anxiety. Within‐group effects were small but significant. The mean effect of depression was 0.06 (95% CI, 0.014 to 0.107; *p* = .011, *Q* = 3.06, *I* ^2^ = 0%) and for anxiety it was 0.08 (95% CI, 0.11 to 0.158; *p* = .024, *Q* = 23.39, *I* ^2^ = 44%). When directly comparing the effect of interventions on anxiety versus depression, between‐group differences were not significant (*Q* = 0.31, *df* = 1, *p* = .578). Subgroup analysis for type of intervention included 8 studies implementing targeted and 13 studies implementing whole‐school interventions. Within‐group effects were small and significant only for studies implementing whole‐school interventions. The mean effect of targeted interventions was 0.01 (95% CI, −0.094 to 0.109; *p* = .88, *Q* = 7.52, *I* ^2^ = 7%) and for whole‐school interventions it was 0.08 (95% CI, 0.036 to 0.117; *p* < .001, *Q* = 12.16, *I* ^2^ = 1%). However, when directly comparing the effect of targeted with whole‐school interventions, between group differences were not significant (*Q* = 1.53, *df* = 1, *p* = .215). Subgroup analysis for age/grade level included 13 studies targeting primary school children and 9 studies targeting secondary school adolescents. Within‐group effects were small and significant only for studies targeting primary schools. The mean effect of primary schools was 0.07 (95% CI, 0.023 to 0.109; *p* = .002, *Q* = 12.61, *I* ^2^ = 5%) and for secondary schools it was .06 (95% CI, −0.014 to 0.144; *p* = .107, *Q* = 8.81, *I* ^2^ = 9%). When directly comparing the effect of primary with secondary school interventions, between‐group differences were not significant (*Q* = 0, *df* = 1, *p* = .979). Lastly, we conducted an additional analysis to examine the effects of methodological quality. Ten studies reporting low risk of bias were compared with 12 studies reporting some to high risk of bias. The mean effect of studies with a low risk of bias was 0.06 (95% CI, 0.014 to 0.113, *p* = .012, *Q* = 12.75, *I* ^2^ = 29%) and for studies with some‐to‐high concerns, 0.07 (95% CI, −0.015 to 0.148, *p* = .110, *Q* = 8.72, *I* ^2^ = 0%). There was no statistically significant difference between low‐risk studies and higher‐risk studies (*Q* = 0.005, *df* = 1, *p* = .946).

### Publication bias

Overall, results were robust to sensitivity analysis examining heterogeneity and publication bias. No outliers were detected using leave‐one‐out analysis and influence diagnostics. Eggers’ test did not indicate presence of funnel plot asymmetry (intercept, 0.073; 95% CI, −0.88 to 0.73; *p* = .861). The SMDs adjusted for publication bias according to Duval and Tweedie trim‐and‐fill procedure barely changed (SMD, 0.067; 95% CI, 0.0282 to 0.1051; *p* < .001). The funnel plot is shown in Figure S2.

### Metaregression analysis of moderators

We assessed the effect of several moderators separately (determined a priori) on internalizing effect sizes using metaregression analysis. Intervention intensity (β = .05; *p* = .303, *R* ^2^ = 14.95%), duration (β = .05; *p* = .312, *R* ^2^ = 5.23%), location (β = −.03; *p* = .439, *R* ^2^ = 0%), and number of intervention components (β = 0.002; *p* = .701, *R* ^2^ = 0%) were not significantly related to or did they account for significant variation in overall internalizing effect sizes. Results of the metaregression analysis are presented in Table S4.

### Metaregression analysis of intervention components

Intervention components were tested separately using metaregression analysis. Inclusion of the component, work with peers (β = .087; *p* = .016, *R* ^2^ = 100%), was significantly associated with a greater reduction in internalizing outcomes and the component cognitive‐behavioral (CBT) elements (β = −0.08; P =.029, R^2^ = 100%) were significantly associated with an increase in internalizing outcomes. No other components were significantly related to, or did they account for significant variation in, overall internalizing effect sizes. Results of the metaregression analysis are presented in Table S5.

### Mediation analysis

Mediation using path analysis was conducted to examine whether reductions in bullying victimization and perpetration mediated intervention effects on overall internalizing symptoms. We found the indirect effect of victimization mediating intervention status on internalizing symptoms (*b* = .058, *p* = .314), and the direct effect of intervention status on internalizing symptoms (*b* = .046, *p* = .189) to be insignificant, implying no mediation effects. We found similar effects with bullying perpetration; the indirect effect of perpetration mediating intervention status on internalizing symptoms (*b* = − .001, *p* = .934) and the direct effect of intervention status on internalizing (*b* = .057, *p* = .052) were also insignificant. Diagrams for both path analyses can be found in Figures S3 and S4.

## Discussion

This systematic review aimed to assess the impact of school‐based antibullying interventions on internalizing symptoms in children and adolescents. A meta‐analysis with 22 studies found a very small but statistically significant effect on overall internalizing symptoms, favoring interventions compared to controls. Sensitivity analyses and adjustments for quality of studies and bias demonstrated that the overall effect size was robust. Subgroup analyses exploring the effect of interventions on anxiety and depression also found a very small but significant effect. Similar results were found for interventions targeting children in primary versus adolescents in secondary school and for types of interventions using targeted versus whole‐school approaches. Despite the effects being statistically significant, it is unclear whether the size of the effects can be considered to be clinically meaningful. Other universal school‐based interventions have commonly powered their effect sizes on mental health outcomes being meaningful at around 0.2–0.3 (e.g. Challen, Machin, & Gillham, 2014; Ford et al., 2012; Gillham et al., 2007; Stallard et al., 2014), whereas the mean effect across both universal and targeted bullying interventions on internalizing symptoms found here was only 0.06. To ensure antibullying trials are maximizing their effectiveness, we recommend researchers power their studies to detect meaningful effects on mental health outcomes.

Our results partially concur with previous systematic reviews. Reviews looking at primary outcomes in antibullying interventions have shown small‐to‐modest reductions in bullying victimization and perpetration (Ferguson, Miguel, Kilburn, & Sanchez, 2007; Merrell, Gueldner, Ross, & Isava, 2008) making it likely that even smaller effects would be observed for secondary outcomes. Likewise, similar to our subgroup analysis, it appears that other meta‐analyses have found inconclusive evidence supporting the effectiveness of other educational setting‐based interventions at preventing anxiety and depression in young people (Caldwell et al., 2019). It is likely that larger (yet still small) effects observed in a previous meta‐analysis are the result of using broader definitions of mental health (Fraguas et al., 2021).

A rather surprising result came from the subgroup analysis examining differences between whole‐school and targeted approaches. We found no difference between intervention types; however, whole‐school approaches were significantly more effective than usual school practice and had a larger effect size than targeted interventions, which were not significantly more effective than usual practice. We had expected targeted interventions to have a more substantial effect on internalizing symptoms as they are typically more effective at managing anxiety and depression than universal interventions (Calear & Christensen, 2010; Werner‐Seidler, Perry, Calear, Newby, & Christensen, 2017) and most only include pupils with high internalizing symptoms at baseline. It may be the case that whole‐school interventions have an advantage because they reach more pupils and often target a wider range of prosocial behaviors and the school climate. Changes in these factors may ultimately help children deal with interpersonal conflicts, foster better relationships, feel safer at school, and thus reduce internalizing symptoms. Indeed, some research suggests that whole‐school interventions can have more successful outcomes when they are integrated into daily practice and school culture, engage all staff and students and are reinforced outside the classroom by parents and the community (Goldberg et al., 2019). Evidence also suggests that a whole‐school approach compared to individual‐ or classroom‐based target interventions is more likely to result in ‘enduring positive changes’ due to the duration and multicomponent focus of these interventions (Weare & Nind, 2011). However, it has been suggested that embedding targeted interventions within whole‐school approaches could be the most effective way to target bullying and potentially internalizing symptoms as this combination would improve overall school climate but also focus on the most vulnerable children and persistent cases of bullying (Garandeau & Salmivalli, 2019). Indeed, more research is needed to untangle the advantages and disadvantages of whole‐school versus targeted approaches. It is clear from this systematic review that very few studies, particularly targeted interventions, applied consistent approaches and targeted similar mechanisms of action, thus potentially contributing to the lack of significant effects. Lastly, it is also possible that different types of interventions had symptom‐specific effects which were not captured with overall internalizing symptoms. Based on our findings, existing targeted antibullying interventions may not be sufficiently tailored to reduce internalizing symptoms, and thus could benefit from re‐examining program aims, content, and design or potentially embedding them in whole‐school interventions to improve student’s mental health outcomes.

We also found that interventions were equally effective at reducing internalizing symptoms for children and for adolescents. Findings from previous literature have been inconsistent in relation to the moderating effects of age on the effectiveness of antibullying interventions on reducing bullying outcomes (Smith, Salmivalli, & Cowie, 2012), however, less is known about its moderating effect on internalizing outcomes. A recent review showed that educational intervention effects on internalizing symptoms were not significantly moderated by age (Werner‐Seidler et al., 2017). To understand this relationship further, age should be examined in combination with program components, type of intervention, type of internalizing outcome, and bullying outcomes.

In terms of intervention components, our findings show that interventions that included a work with peers component were associated with larger effects on internalizing symptoms than those that did not use this component. Recent reviews show that work with peers is associated with a decrease in victimization (Gaffney et al., 2021a, 2021b); however, this is the first time it has been looked at in association with internalizing outcomes. Working with peers has been shown to increase student’s perception and sense of safety at school (Cowie, Hutson, Oztug, & Myers, 2008; Cowie & Oztug, 2008), which may ultimately lead to overall reductions in internalizing symptoms. Some of the most effective interventions at reducing bullying such as KiVA (Salmivalli, Poskiparta, Ahtola, & Haataja, 2013) and No Trap! (Palladino, Nocentini & Menesini, 2016) involve working with peers in different capacities (e.g. as mediators or by encouraging bystander behavior) and further research should investigate which specific capacities are the most helpful at reducing internalizing symptoms. For example, in a recent review by Gaffney et al. (2021a, 2021b), researchers explored the distinction between informal and formal peer involvement in addition to encouraging bystander behavior for students witnessing bullying. Results showed that informal peer involvement was associated with significant reductions in bullying perpetration and victimization, while formal peer involvement was associated with significant reductions in bullying perpetration only. Surprisingly, the *exclusion* of encouraging bystander behavior was associated with significantly larger reductions in victimization. The difference in results for each, albeit nonmutually exclusive, category shows the need to make these distinctions to try and understand what makes interventions work better. Notably, in past systematic reviews, work with peers had been found to be associated with an *increase* in victimization, and it has been suggested that this might be the case if peer leaders have high levels of externalizing symptoms which might lead to deviancy training and negative outcomes (Dishion, McCord, & Poulin, 1999). Therefore, future work examining baseline levels of internalizing and externalizing in peer leaders, and other potential moderators, should be considered when evaluating program effects on victimization and mental health.

Contrary to expectations, given that some studies suggest that components targeting mental health (including CBT) can be effective at reducing bullying perpetration (Gaffney et al., 2021a, 2021b), the use of CBT components was significantly associated with *smaller* effects on internalizing symptoms compared to when CBT components were not used. This negative effect appeared to be carried by two of the seven interventions which used CBT. These were both targeted interventions: one delivered general counseling to children in care and the other taught emotional literacy to children identified as bullies by their peers. However, there was no clear pattern in outcomes based on study type or program duration and intensity. Also of note, most of the interventions that implemented CBT components were delivered by school staff; research on programs targeting internalizing symptoms suggests that larger effect sizes are associated with programs delivered by mental health professionals than programs delivered by teachers (Calear & Christensen, 2010; Stallard et al., 2014; Werner‐Seidler et al., 2017). Our findings raise reasons to be cautious about the use of CBT for dealing with internalizing symptoms in antibullying contexts and support other reviews showing similar trends from other school‐based interventions (Caldwell et al., 2019; Mertens, Deković, Leijten, Van Londen, & Reitz, 2020). Yet, future research should examine whether incorporating specific CBT techniques can potentially promote children’s mental health in antibullying interventions, depending on study type, design, their use in combination with other components (e.g. work with peers), their frequency, and their mode of delivery.

We found no evidence that other components examined in this review were effective in reducing internalizing symptoms, but this might reflect the small number of studies that applied consistent approaches and targeted similar mechanisms of action. For example, some universal interventions were based on restorative practice (Bonell et al., 2018) or adventure‐based learning (Battey, 2008), while targeted programs varied among CBT‐based, personality‐targeted approaches (Kelly et al., 2020), social skills training (Fox & Boulton, 2003), or art therapy (Yan, Chen, & Huang, 2019). Going forward it will be important to examine the specific impacts of components grounded in empirical knowledge and theory of change frameworks, both to maximize meaningful clinical impacts and to provide a deeper understanding of any causal pathways between bullying and internalizing symptoms. Lastly, although we examined each intervention component individually, it is important to note that we did not assess for fidelity, and thus future studies should consider measuring adherence to interventions components. It is likely that factors such as the time required for preparation of lessons, duration of activities, and teacher’s available time and enthusiasm for individual components throughout the intervention, among other factors, may influence program fidelity.

Our last set of analyses examined the mediating effects of bullying with the aim of exploring a possible causal pathway for intervention effects. Reductions in bullying victimization and perpetration did not account for intervention effects on internalizing outcomes. It is possible that antibullying interventions may be acting in many other ways to reduce internalizing, such as by promoting a positive school climate, encouraging bystanders to take action, fostering prosocial skills, through peer support or other types of social support, or possibly a combination of elements that work towards improving student’s mental health. To our knowledge, this is the first study to investigate potential causal pathways in a meta‐analysis of antibullying interventions and sets a framework for prospective research to explore other factors that might explain intervention mechanisms and target them in future interventions designed to decrease bullying and internalizing symptoms. It is important to note that despite using pre–post changes for this analysis to account for baseline levels of bullying and internalizing, small effects and low heterogeneity could have had an impact on this analysis. Furthermore, this analysis only reflects school‐level and not student‐level mediation effects.

### Limitations and future directions

Our findings should be interpreted in the context of several limitations. In terms of our included studies, quality and risk of bias varied widely. Studies typically scored high on risk of bias due to no randomization taking place, data handling being inadequate, or missing data and attrition not being described. Yet, subgroup analyses looking at overall study quality showed no difference between low and high risk of bias. In terms of statistical analysis, many studies did not report the necessary statistics to compute effect sizes and thus were excluded from the meta‐analysis. Additionally, most of our outcomes were based on self‐report which could inflate the risk of bias. Unfortunately, we were unable to test type of informant as a moderator due to a lack of studies with informants other than self‐reports. In terms of intervention components, we did not preregister or distinguish between specific types of the ‘work with peers’ component (e.g. Gaffney et al., 2021a, 2021b) as this could lead to a loss of power and potentially biased results.

There are also other limitations to consider that are specific to this review. Our objective was to examine interventions with a primary aim to reduce bullying in schools. As such, we excluded general conflict resolution and aggression prevention interventions as well as general health promotion interventions which could have an impact on both bullying and internalizing symptoms. This review focused on internalizing symptoms as outcome measures as they are the leading cause of poor mental health in children (Erskine et al., 2015; Ghandour et al., 2019). We recognize that antibullying interventions may also have an impact on other outcomes such as externalizing symptoms, and to a lesser extent general wellbeing. Although we tried to capture a range of internalizing and emotional symptoms measured in a standardized and validated way, we excluded studies if they only presented measures of self‐esteem, loneliness, or guilt as we did not consider them to be fully representative of internalizing symptoms. Standardization of measures should be considered for future intervention studies to promote data synthesis and comparison of findings across studies.

In terms of statistical analysis, there were also some limitations. First and foremost, having average effect sizes to obtain a single measure of internalizing may have resulted in a loss of information for metaregression analyses, particularly in cases where depression and anxiety had opposing effects, or if one outcome was significantly different from the other. Effects were computed at postintervention only, however, it is unlikely that results would drastically change over time as seen in previous reviews looking at intervention effects on anxiety and depression (Werner‐Seidler et al., 2017). Other statistical limitations included having to control for clustering with studies that did not report intraclass correlation coefficients (ICC). In these cases, ICCs were drawn from the literature, but might not be fully representative of specific trials. Lastly, it is likely that our lack of heterogeneity contributed to our secondary analysis observing minimal effects.

## Conclusion

Overall, we found insufficient evidence to conclude that antibullying interventions are effective at reducing internalizing problems in children and adolescents. While we are confident that most antibullying programs are effective at reducing the prevalence of bullying victimization and perpetration in schools, this review suggests that more work needs to be done to maximize the mental health benefits of these interventions. Specifically, there is a need to select and measure internalizing outcomes consistently, power studies to be able to detect clinically significant changes in internalizing effect sizes, improve the quality of research designs, and design antibullying interventions using evidence‐based components that are likely to enhance positive impacts on mental health. This review suggests that including social or peer support components may be effective at reducing internalizing symptoms and therefore could be a target for intervention. On the other hand, interventions should carefully re‐evaluate how CBT components are being employed to optimize their effect. Moving forward, research should prioritize understanding the causal pathways between intervention effects and mental health outcomes to highlight the specific type of support needed in interventions. Targeting internalizing outcomes in schools offers the opportunity to alleviate many typical barriers to accessing treatment such as time, location, and cost. Thus, future researchers, clinicians, and public healthcare professionals should recognize the importance of internalizing outcomes when designing school‐based interventions to maximize their impact and safeguard young people from the damaging outcomes of bullying.

## Supporting information


**Appendix S1.** Note on deviations from the Protocol.
**Appendix S2.** Detailed Search Strategy.
**Appendix S3.** Intervention component definitions.
**Table S1.** Rating of intervention components.
**Table S2.** Study and participant characteristics.
**Table S3.** Characteristics associated with study design.
**Table S4.** Moderator Metaregression analysis.
**Table S5.** Intervention components Metaregression analysis.
**Figure S1.** Quality assessment (RoB).
**Figure S2.** Duval and Tweedie Trim‐and‐Fill Funnel Plot.
**Figure S3.** Mediation model for Bullying Victimization.
**Figure S4.** Mediation model for Bullying Perpetration.
