# Supplementary material for: Research Review: Do antibullying interventions reduce internalizing symptoms? A systematic review, meta‐analysis, and meta‐regression exploring intervention components, moderators, and mechanisms
Source: J Child Psychol Psychiatry. 2022 Apr 26;63(12):1454–65. doi: 10.1111/jcpp.13620 (PMC9790441; doi:10.1111/jcpp.13620)
Supplement: Supplementary file 1 — Appendix S1. Note on deviations from the Protocol. Appendix S2. Detailed Search Strategy. Appendix S3. Intervention component definitions. Table S1. Rating of intervention components. Table S2. Study and participant characteristics. Table S3. Characteristics associated with study design. Table S4. Moderator Metaregression analysis. Table S5. Intervention components Metaregression analysis. Figure S1. Quality assessment (RoB). Figure S2. Duval and Tweedie Trim‐and‐Fill Funnel Plot. Figure S3. Mediation model for Bullying Victimization. Figure S4. Mediation model for Bullying Perpetration. [file JCPP-63-1454-s001.docx]

**Supporting Information**

1. Appendix S1. Note on deviations from the Protocol
2. Appendix S2. Detailed Search Strategy
3. Appendix S3. Intervention Component Definitions
4. Table S1. Rating of Intervention Components
5. Table S2. Study and Participant Characteristics
6. Table S3. Characteristics associated with study design
7. Table S4. Moderator Meta Regression analysis
8. Table S5. Intervention Components Meta Regression analysis
9. Figure S1. Quality Assessment (RoB)
10. Figure S2. Duval and Tweedie trim-and-fill Funnel Plot
11. Figure S3. Mediation model for Bullying Victimization
12. Figure S4. Mediation model for Bullying Perpetration
13. **Appendix S1. Note on deviations from the protocol**

First and foremost, we made the decision to use Hedge’s *g* instead of Cohen’s *d* to control for overestimation biases in small sample sizes (Hedges, 1981). Secondly, it was our intention to use internalizing post-scores for moderation and mediation analysis, before realizing that it would be impossible to standardize the measures as there was substantial variability in the measures used in each study. As a result, we opted to use effect size measures (Hedge’s g) as our outcome measure. Thirdly, for mediation analysis, we used mean pre-post effect sizes for internalizing and bullying outcomes. Additionally, we opted to include 2 further exploratory sub-group analysis: type of intervention (targeted vs. whole-school) and quality of study (low bias vs. some/high risk of bias) as we believed it would yield valuable information. Despite deviations to our protocol, the changes made were carefully considered and determined to improve the quality of the study and reduce biases.

1. **Appendix S2. Detailed Search Strategy**

The same keywords were used for each database and searched in titles and abstract fields (or equivalent), with the exception of line 3 which was searched in full-texts as secondary outcomes may not come up in titles or abstracts. The following search terms were used:

1. “intervention*” OR “program*” OR “anti-bullying” OR “anti-bullying”
2. “bulli*” OR “bully*” OR “victim*”
3. “internali#ing” OR “depress*” OR “anxi*” OR “somati*” OR “withdraw*” OR “emotional functioning” OR “emotional symptom*” OR “emotional problem*” OR “emotional difficult*”
4. “adolescen*” OR “child*” OR “school*” OR “youth”
5. Combine 1 AND 2 AND 3 AND 4.

For our author search, we used the same authors as Ttofi & Farrington (2011): Ken Rigby (Australia), Peter Smith (England), Christina Salmivalli (Finland), Rosario Ortega (Spain), Dan Olweus (Norway).

1. **Appendix S3. Intervention Component Definitions:**

**Whole-school anti-bullying policy:** Incorporates or amends a formal anti-bullying policy/constitution in the school.

**Classroom rules:** rules set out by a class against bullying that they are expected to follow.

**School conference/assembly:** school assemblies talks or conferences where the program is explained and children are informed about bullying.

**Curriculum materials:** the use of anti-bullying material or lessons incorporated into a new or pre-existing curriculum.

**Interactive activities/games:** games included in the intervention such as role-play aimed at demonstrating concepts of bullying

**Cooperative group work among experts:** Working in groups to implement the intervention including different professionals (teachers, psychologists, health care professionals, researchers, etc…)

**Work with bullies and victims:** Individualized work (not at the classroom level) with children involved in bullying as victims or perpetrators. This may include counselling.

**Work with peers:** this involves engaging peers in specific roles such as mentors (e.g. peer training) or mediators (e.g. peer mediators) for bullying situations. This can also involve bystander training.

**Information for parents and teachers:** this would include program manuals or guides that teachers or parents could consult to implement the program, or newsletters to parents about the program.

**Improved playground supervision**

Designating school staff to recess and playground supervision or monitor identified ‘hot-spots’ of bullying.

**Disciplinary methods**

Including any punitive method in dealing with bullying situations

**Non-punitive methods**

Including any non-punitive method in dealing with a bullying situation (e.g. restorative approaches)

**Teacher training:** Any formal workshop, lesson, course or type of training (of any duration and intensity) administered to teachers or school staff about how to implement the program or deal with bullying situations.

**Parent involvement:** involving parents to complete parts of the intervention (e.g. homework assignments) or requesting their presence in organized “information nights” or presentations about the intervention.

**Video/VR/computer games:** Using technology to implement intervention materials or lessons, raise awareness, etc..

**Internalizing symptoms:** Any feature of the program that may be specifically designed to target and improve internalizing symptoms such as anxiety or depression.

**Social skills training:** some programs specifically aim to improve social skills or train children how to cope with bullying through social-emotional learning

**Empathy training:** some programs specifically teach or raise awareness of empathy (in lessons or games)

**Cognitive-behavioural elements:** Programs that include CBT-type components, typically incorporating a psychologist or trained councellor.

**Other:** Any other intervention component.

| **Table S1. Rating of Intervention Components** | | | | | | | | | | | | | | | | | | | | |
| --- | --- | --- | --- | --- | --- | --- | --- | --- | --- | --- | --- | --- | --- | --- | --- | --- | --- | --- | --- | --- |
| Authors | 1 | 2 | 3 | 4 | 5 | 6 | 7 | 8 | 9 | 10 | 11 | 12 | 13 | 14 | 15 | 16 | 17 | 18 | 19 | 20 |
| Battey, 2008 | N | N | N | Y | Y | N | N | N | N | N | N | N | N | N | N | N | N | N | N | N |
| Berry and Hunt, 2009 | N | N | N | N | N | Y | N | N | N | N | N | N | N | Y | N | Y | Y | N | Y | N |
| Bonell et al., 2018 | Y | N | N | Y | Y | Y | N | Y | N | N | Y | N | Y | N | N | N | Y | Y | Y | N |
| Connolly et al., 2015 | N | N | N | Y | N | N | Y | N | N | N | N | N | N | N | N | N | N | N | N | N |
| Cross et al., 2018 | Y | N | N | Y | Y | Y | N | Y | Y | N | Y | N | Y | Y | N | Y | Y | Y | Y | Y |
| Dempsey, 2009 | N | N | N | Y | N | N | Y | Y | N | N | N | N | Y | N | N | N | N | N | N | N |
| DeRosier, 2004 | N | N | N | Y | Y | Y | N | N | N | N | N | N | N | N | N | N | Y | N | Y | N |
| Fox and Boulton, 2003 | N | N | N | N | N | Y | N | N | N | N | N | N | N | N | N | N | Y | N | N | N |
| Hoglund et al., 2012 | N | N | Y | Y | Y | N | N | Y | Y | N | Y | N | Y | N | N | N | Y | N | N | N |
| Huitsing et al., 2019 | Y | Y | N | Y | Y | Y | Y | Y | Y | N | Y | N | Y | Y | Y | N | Y | Y | N | N |
| Pintabona, 2005 | Y | N | N | Y | Y | Y | N | Y | Y | N | Y | N | Y | Y | N | N | Y | Y | Y | Y |
| Rapee et al., 2020* FS | Y | N | N | Y | Y | Y | N | Y | Y | N | Y | N | Y | Y | N | N | Y | Y | Y | N |
| Rapee et al., 2020** CKTC | N | N | N | Y | Y | Y | N | Y | N | N | N | N | N | Y | Y | Y | Y | N | Y | N |
| Rapee et al., 2020*** COMB | Y | N | N | Y | Y | Y | N | Y | Y | N | Y | N | Y | Y | Y | Y | Y | Y | Y | N |
| Yan, Chen and Huang, 2019* | N | N | N | N | N | Y | N | N | N | N | N | N | N | N | N | N | N | N | N | N |
| Yan, Chen and Huang, 2019** | N | N | N | N | N | Y | N | N | N | N | N | N | N | N | N | N | N | N | Y | N |
| Nocentini et al., 2018 | N | Y | Y | Y | Y | Y | Y | Y | Y | N | Y | N | Y | Y | N | N | N | Y | N | N |
| Palladino et al., 2019 | Y | N | Y | N | Y | N | Y | N | N | N | N | N | Y | N | Y | N | Y | Y | Y | N |
| Knowler & Frederickson, 2013 | N | N | N | Y | N | Y | N | N | N | N | N | N | Y | N | N | Y | Y | Y | Y | N |
| Van Ryzin and Roseth, 2018 | N | N | N | N | N | N | Y | Y | N | N | N | N | Y | N | N | N | N | N | N | N |
| Williford et al., 2012 | Y | Y | N | Y | Y | Y | Y | Y | Y | N | Y | N | Y | Y | Y | N | Y | Y | N | N |
| Kelly et al., 2020 | N | N | N | N | N | N | N | N | N | N | N | N | N | N | N | Y | N | N | Y | Y |
| Y = yes; N = no; 1 = whole school policy; 2 = classroom rules; 3 = school conference or assembly; 4 = curriculum materials; 5 = cooperative work among experts; 6 = work with bullies and victims; 7 = work with peers; 8 = information for teachers; 9 = playground supervision; 10 = disciplinary methods; 11 = non-punitive methods; 12 = school tribunals or courts; 13 = teacher training; 14 = parent training or meetings; 15 = video or VR computer games; 16 = internalising symptoms; 17 = social skills training; 18 = empathy training; 19 = CBT; 20 = other. | | | | | | | | | | | | | | | | | | | | |

| **Table S2. Study and participant characteristics** | | | | | | | | |  |
| --- | --- | --- | --- | --- | --- | --- | --- | --- | --- |
| **Study** | **Country** | **Year of Intervention** | **Subjects** | **Grade level (age range or M)** | **Name of Intervention** | **Type of Intervention** | **Duration/Intensity of Intervention** | **Mode of delivery** | **Program Costs** |
| Battey (2008)* | USA | 2007 | 107 students in 2 schools** | 7^th^ (M=12 years) | Bully Prevention Challenge Course Curriculum (BPCCC) | Whole-school (adventure based learning) | 1-2, 45 min classes, 4x per week for 1 week | School staff (e.g. physical education/health teacher) | Not stated |
| Berry and Hunt (2009)* | Australia | 2004 | 46 students in 7 schools** | 7^th^ to 10^th^ (12-15 years) | Confident Kids Program | Targeted (cognitive behavioural group intervention for anxious boys) | 1 hr 8x per week | Intern clinical psychologists | Not stated |
| Bonell et al. (2018)* | England | 2014-2017 | 7154 students in 40 schools*** | 7^th^ to 10^th^ (11-12 years) | Learning Together (INCLUSIVE) Intervention | Whole-school (focuses on changing school environment) | 5-10hrs of lessons per year | Trained teachers | £116 per pupil in the control group compared to £163 per pupil in the intervention group over the first 2 years or £58 and £82 per student per year. |
| Connolly et al. (2015)* | Canada | Not given | 447 students in 4 schools | 7^th^ and 8^th^ (11-14 years) | Respect in Schools Everywhere (RISE) | Whole-school (youth-led aggression prevention program) | Leaders give two 45 min classes (90 min). UP schools had three adult-led programs lasting 90 min. | Trained high school youth in YLP or adults (teacher or councillor) in UP | Not stated |
| Cross et al. (2018)* | Australia | 2006-2007 | 2690 students in 20 schools** | 8^th^ and became 9^th^ for post (M=14 years) | Friendly Schools Project | Whole-school (socio-ecological approach with parent component) | 6 hours for Grade 8 students and 3.5 hours for Grade 9 students. | Teachers and school staff | Not stated |
| Dempsey and Waldron (2009)* | USA | 2007-2008 | 283 students in 4 schools** | 6^th^ to 8^th^ | The Aggressors, Victims and Bystanders (AVB) curriculum | Whole-school (curriculum based using 4-step conflict resolution model) | Curriculum delivered over 12 weekly sessions from October to January | Teachers | Not stated |
| DeRosier (2004)* | USA | Not given | 381 students in 11 schools*** | 3^rd^ to 5^th^ (7-11 years) | S.S. Grin | Targeted (social learning and cognitive-behavioural techniques for anxious children with peer difficulties) | Group sessions lasted 50 to 60 min and took place once weekly for 8 weeks. | School councillor and undergraduate intern | Not stated |
| Fekkes, Pijpers and Verloove-Vanhorick (2006) | Netherlands | 1999-2001 | 1591 students from 33 schools** | “upper 3 grades” (9-12 years) | Antibullying school program in the Netherlands | Whole-school (involves teacher training, parental component and development of anti-bullying curriculum) | Not specified; lasting one year | Teachers | Not stated |
| Finger (2018) | Australia | 2006-2007 | 681(W3), 685(W4), 673(W5) students in 6 schools | 5^th^ to 6^th^ | Beyond Bullying Primary School Program | Whole-school (ecological model targeting “school culture, antibullying policy, teacher management, student action and parental involvement”) | Intensity not specified but lasted 10 weeks | Researchers (PhD student) | Not stated |
| Fite et al. (2019) | USA | 2015-2016 | 24 students in 1 school** | 2^nd^ to 4^th^ grade at baseline and 3^rd^ to 5^th^ for intervention (7-11 years) | Taking Action | Targeted (focuses on problem solving, coping skills, cognitive restructuring for bullied children) | 21–24 group sessions held for approximately 30 min each, twice a week | Researchers | Not stated |
| Fox and Boulton (2003)* | England | 1999-2000 | 28 students in 4 schools** | 4^th^ to 6^th^ (aged 9-11 years) | The Social Skills Training Programme | Targeted (social-emotional learning approach for bullied children) | 1 hr/week x 8 weeks | Teachers and school staff | Not stated |
| Hoglund, Hosan and Leadbeater (2012)* | Canada | 2000-2003 | 432 students in 17 schools*** | 1^st^ to 3^rd^ (M=6 years) | The WITS Primary Program | Whole-school Intervention (curriculum based) | Duration or intensity not specified but lasting 3 years | Teachers | Not stated but WITS materials are available for free. |
| Huitsing et al. (2019)* | Netherlands | 2012-2013 | 4356 students in 99 schools*** | 3^rd^ and 4^th^ (dutch grades 5^th^ and 6^th^), (M=9 years at FU) | KiVa | Whole-school intervention (curriculum based, raising empathy and altering bystander behaviour) – **based on a target sample** | Not specified | Teachers | Not stated, but other papers suggest the cost for implementing KiVa is about €21 or £18 per student per year, not including annual and once-only costs. |
| Karmaliani et al. (2020) | Pakistan | 2015-2018 | 1598 students in 40 schools*** | 6^th^ to 8^th^ grade (9-15 years) | Right to Play Intervention | Whole school intervention (structured play-based intervention) | 120 sessions (2 35 min sessions per week) for 2 years | Collaborative Coaches employed by NGO and junior leaders | Not stated |
| Kelly et al. (2020)* | Australia | 2012 | 2190 students in 26 schools*** | 7^th^ to 10^th^ (M=13 years) | Climate and Preventure | Combines a universal ‘Climate’ and targeted ‘Preventure’ program - a brief CBT-based personality-targeted intervention) | Two 90-minute group sessions, 1 week apart. 162 sessions were completed between March and November 2012. | Psychologist and other trained facilitators | Not stated |
| Knowler and Frederickson (2013)* | England | Not given | 50 students in 4 schools | 3^rd^ to 5^th^ (8-9 years) | Social and Emotional Aspects of Learning (SEAL)/ | Targeted (for children engaging in bullying behaviours, based on teaching emotional literacy) | 12 weekly 45-60 min sessions | Teachers | Not stated |
| Nocentini, Menesini and Pluess (2018)* | Italy | 2013-2014 | 2042 students in 13 schools*** | 4^th^ to 6^th^ (M=10 years) | KiVa | Whole-school (curriculum based, raising empathy and altering bystander behaviour) | Not specified but lasting less than a year | Teachers | Not stated |
| Palladino, Benedetta, Nocentini & Menesini (2019)* | Italy | 2011-2012 | 622 students in 8 schools** | 9^th^ (M=15 years) | No Trap! | Whole-school (revise antibullying policy, teach awareness, nominate peer-educators to deliver activities and moderate online social media) | Two 2hrs classes of awareness; two 2hr meetings where face to face peer educators did activities with their own class | Teachers and peers | Not stated |
| Parada, Craven and Marsh (2008) | Australia | Not given | 5204 students in 6 schools** | 7^th^ to 11^th^ | Beyond Bullying Secondary Program | Whole-school (building antibullying school policy) | Not specified but lasting 2 years | Teachers | Not stated |
| Pintabona (2005)* | Australia | 2000-2001 | 296 students in 29 schools** | 4^th^ (M=8 years) | Friendly Schools Project | Whole-school (curriculum-based with family component) | 9 hours of lessons (three units of 3 x 60 min activities presented across 3 school terms). | Teachers and school staff | Not stated |
| Pryce and Frederickson (2013) | England | Not given | 338 students in14 schools | 4^th^ to 6^th^ (8-11 years) | Antibullying Pledge Scheme (ABPS) | Whole-school (curriculum-based, universal prevention program) | Not specified | Teachers and educational psychologist | Not stated |
| Rahey and Craig (2002) | Canada | Not given | 491 students in 2 schools | 1^st^ to 8^th^ grade | Bully Proofing your school | Whole school program | 12 weeks (individual sessions 45 min each week, 3 teacher sessions) | Teachers and researcher graduate students | Not stated |
| Rapee et al (2020)* | Australia | 2015-2016 | 7701 students in 135 schools** | 3^rd^ and 4^th^ (7-12 years) | Friendly Schools (FS); Cool Kids Taking Control (CCTK); Combination | Whole-school (FS) with Targeted (CCTK) for children who are currently being victimised and have high symptoms of anxiety | FS includes 15 activities (∼40 min). CKTP parents were given lesson weekly for 8 weeks. | Teachers and school staff; parents (for targeted intervention) | Not stated |
| Van Ryzin and Roseth (2018)* | USA | 2016 | 1460 students in 15 schools*** | 7^th^ | Johnson’s Cooperative Learning Approach | Whole-school (uses cooperative learning and peer-led activities) | Not specified | Teachers | Not stated |
| Williford et al. (2012)* | Finland | 2007-2008 | 7741 students in 78 schools*** | 3^rd^ to 5^th^  at baseline, 4^th^ to 6^th^ during intervention (M=11 years) | KiVa | Whole-school (curriculum based, raising empathy and altering bystander behaviour) | 20 hrs of curriculum | Teachers and school staff | Not stated, but other papers suggest the cost for implementing KiVa €43 or £37 per student per year, not including annual and once-only costs. |
| Yan, Chen and Huang (2019)* | China | 2017-2018 | 169 students in 6 schools** | 5^th^ (M=11 years) | Art Intervention vs. general counselling | Targeted (art therapy vs. general counselling for LBC victims of bullying) | Art therapy and counselling conducted in 6 sessions over 3 months. | Teachers | Not stated |
| Zagorscak et al. (2019) | Germany | 2011 | 722 students in 5 schools** | 7^th^ to 10^th^ (11-17 years) | Media Heroes | Whole-school (short and long format – curriculum based) | IG-L (90 min session per week for 10 weeks); IG-S (4 sessions of 90 min in one day) | Teachers | Not stated |

*Author = included in meta-analysis

**Sample at last time point

***Analyzed sample

| **Table S3. Characteristics associated with study design** | | | | |
| --- | --- | --- | --- | --- |
| **Study** | **Study Design** | **Measure of Internalizing** | **Time between assessments** | **Quality rating (risk of bias)** |
| Battey (2008) | Quasi-experimental, non-randomised, pre-post with control | CES-DC (depression) | Post: after each class FU: 3mo | High |
| Berry and Hunt (2009) | CRCT | CES-DC (depression) SCARED (anxiety) | Post: 8weeks  FU: 3mo | Low |
| Bonell et al. (2018) | CRCT | SDQ (emotional problems subscale) | Post: 24months  FU: 36 months | Low |
| Connolly et al. (2015) | CRCS | SCARED (anxiety) | Pre: October  Post: 7mo | Some concern |
| Cross et al. (2018) | CRCT | DASS (depression and anxiety) | Pre: start of grade 8  Post: end of grade 8  FU: end of grade 9 | Some concern |
| Dempsey and Waldron (2009) | Quasi-experimental, non-randomised, pre-post with control | CES-DC (depression)  SASA (social anxiety) | Pre: 4-weeks after classes started (Sept) and 1-weeks before curriculum delivery  Post: 4mo (Feb) | High |
| DeRosier (2004) | IRCS | MFQ (depression)  SASC-R (social anxiety) | Pre: October  Post: 6mo (April)  FU: 1yr (April) | Low |
| Fekkes, Pijpers and Verloove-Vanhorick (2006) | CRCS | SDI-C (depression) Psychosomatic complaints (not a scale) | Pre: Nov 1999  Post: 6mo (May 2000)  FU: 12mo (May 2001) | Low |
| Finger (2018) | Quasi-experimental, non-randomised, cross-over with control | SDQ-IE (emotional stability subscale)  CDI (depression) | Baseline: w1 (Feb); w2 (Aug)  Experimental: w3 (Feb), w4 (Aug), w5 (Nov) | High |
| Fite et al. (2019) | Quasi-experimental, non-randomised, pre-post with control | SMFQ (depression) Teacher Report Form (withdraw/depressed)  PROMIS (anxiety) | Pre: Fall 2016  Post: Spring 2017 | High |
| Fox and Boulton (2003) | Quasi-experimental, non-randomised, pre-post with control | RCMAI (anxiety)  CDI (depression) | Post:3mo  FU: 6mo | High |
| Hoglund, Hosan and Leadbeater (2012) | Quasi-experimental, non-randomised, pre-post with control | ESBS (internalising, used W1-4)  BAS-C (internalising used W 5-6) | Pre: Fall of grade 1  Post & FU: Springs of grade 2,3,5 and 6 (WITS was implemented grades 1-3) | High |
| Huitsing et al. (2019) | CRCT | 9-item scale derived from an MDD scale (depression)  SPQ (social anxiety) | Pre: May 2012 or in T2 (Oct 2013) if measurement not available  Post: Oct 2012  FU: 1yr (May 2013) | Low |
| Karmalani et al. (2020) | CRCT | CDI-2 (depression) | Pre: 2015  Post: 2 years (2017-18) | Low |
| Kelly et al. (2020) | CRCT | SDQ (emotional problems subscale) | Post: 12mo  FU: 24mo, 36mo | High |
| Knowler and Frederickson (2013) | IRCS | SDQ (emotional problem subscale) | Post: 12weeks | High |
| Nocentini, Menesini and Pluess (2018) | CRCT | YSR (internalising behaviours) | Pre: Sept to Oct 2013  Post: May to June 2014 | Low |
| Palladino, Benedetta, Nocentini & Menesini (2019) | Quasi-experimental, non-randomised, pre-post with control | YSR (internalising behaviours) | Pre: Nov 2011  Mid: Feb 2012 (only adult-led not peer-led)  Post: May-June 2012 | High |
| Parada, Craven and Marsh (2008) | Quasi-experimental, non-randomised, cross-over with control | SDQ-II (emotional stability subscale)  CDI (depression) | Baseline: T1 (March), T2 (July), T3 (Dec)  Pre (BL collection): T4 (April YR 2)  Post: T5 (Aug), T6 (Dec) | Some concern |
| Pintabona (2005) | CRCT | CDI (depression)  RCMAS (anxiety)  BASC-PR (depression, anxiety and somatic symptoms) | Post: 12mo  FU: 16mo, 32mo | Low |
| Pryce and Frederickson (2013) | Quasi-experimental, non-randomised, pre-post with control | SDQ (emotional problems subscale) | Post: 2-3mo | High |
| Rahey and Craig (2002) | Quasi-experimental, non-randomised, pre-post with control | CBC (internalising subscales) | Post = 12 weeks | Some concern |
| Rapee et al (2020) | CRCT | SCAS (anxiety)  MFQ (depression) | Post: 12mo  FU: 24 mo (from baseline) | Low |
| Van Ryzin and Roseth (2018) | CRCT | SDQ (emotional problems subscale) | Pre: Sept/Oct 2016  Post: 5.5mo (March 2017) | Some concern |
| Williford et al. (2012) | CRCT | BDI (depression)  FNE (social anxiety)  SADS (social anxiety) | Pre: May 2007  Post: Dec 07/Jan 08  May: 2008 | Low |
| Yan, Chen and Huang (2019) | IRCS | SASC-R (social anxiety) | Pre: June 2017 – April 2018  Post: not specified | Some concern |
| Zagorscak et al. (2019) | CRCT | Bern Wellbeing Questionnaire for Adolescents (somatic complaint scale) | Pre: Jan 2011  Post: 9mo (6mo after intervention, Nov/Dec 2011) | High |

CRCT/s = Cluster Randomised Controlled Trial/Study; IRCT/s = Indiviual Randomised Controlled Trial/Study; CES-DC = The Center for Epidemiological Studies Depression Scale for Children; SCARED = Screen for Child and Anxiety Related Disorders; DASS = Depression Anxiety Stress Scale; CDI= Child Depression Inventory; RCMAS = Revised Children’s Manifest Anxiety Scale; RCMAI = Revised Children’s Manifest Anxiety Inventory; BASC-PR = Behaviour Assessment System for Children Parent Rated; YSR = Youth Self-Report Scale; SPQ = Social Phobia Questionnaire; SCAS = Spence Children’s Anxiety Scale; MFQ = Mood and Feelings Questionnaire; SMFQ = Short Mood and Feelings Questionnaire; PROMIS = Patient‐Reported Outcomes Measurement Information System; SDQ = Strengths and Difficulties Questionnaire; SDQ-II= Self-Description Questionnaire; SASC-R = Social Anxiety Scale for Children Revised; SASA = Social Anxiety Scale for Adolescents; BDI = Beck Depression Inventory; RBDI = Beck Depression Inventory Short Version; SDI-C = Short Depression Inventory for Children; FNE = Fear of Negative Evaluation Scale; SADS = Social Avoidance and Distress Scale; CBC = Child Behaviour Checklist; ESBRS = Early School Behaviour Rating Scale; BAS-C = Behavioural Assessment Scale for Children

| **Table S4. Meta-regression Analysis of Moderators** | | | | | | | | | |
| --- | --- | --- | --- | --- | --- | --- | --- | --- | --- |
| **Moderator** | **B** | **SE** | **P value** | **95% CI lb** | **95% CI ub** | **R^2^** | **I^2^** | **Test for Residual Heterogeneity** | **Test of Moderators** |
| **Intensity:** Intensity of intervention in less than 10 hours or more than/equal to 10 hours in total | 0.0452 | 0.0439 | 0.3032 | -0.0408 | 0.1312 | 14.95 | 2.76 | Q_E_(df= 18) = 18.51, p = 0.422 | Q_M_(df=1) = 1.06, p = 0.303 |
| **Duration**: Duration of intervention is less than 12 months or more than/equal to 12 months | 0.0485 | 0.0480 | 0.3124 | -0.0456 | 0.1427 | 5.23% | 2.04% | Q_E_(df= 20) = 20.42, p= 0.432 | Q_M_(df=1) = 1.02, p = 0.312 |
| **Location:** By region: North America, Asia and the Pacific or Europe | -0.0292 | 0.0377 | 0.4387 | -0.1031 | 0.0447 | 0% | 3.77% | Q_E_(df= 20) = 20.78, p = 0.410 | Q_M_(df=1) = 0.600, p = 0.439 |
| **Number of Intervention Components:**  Sum of all components implemented in the intervention, independent of fidelity. | 0.0020 | 0.0052 | 0.7005 | -0.0082 | 0.0123 | 0% | 5.93% | Q_E_(df= 20) = 21.26, p = 0.382 | Q_M_(df=1) = 0.148, p = 0.701 |

| **Table S5. Intervention components meta-regression analysis** | | | | | | | | | |
| --- | --- | --- | --- | --- | --- | --- | --- | --- | --- |
| Component | B | SE | P value | 95% CI lb | 95% CI ub | R^2^(%) | I^2^(%) | Test for Residual Heterogeneity | Test of Moderators |
| Whole-School Antibullying policy | -0.002 | 0.037 | 0.973 | -0.0870 | 0.0841 | 0 | 6.86 | Q_E_(df = 20) = 21.47, p = 0.370 | QM(df = 1) = 0.00, p = 0.973 |
| Classroom rules | 0.065 | 0.037 | 0.078 | -0.0074 | 0.1371 | 100 | 0 | QE(df = 20) = 18.38, p-val = 0.563 | QM(df = 1) = 3.10, p-val = 0.078 |
| School conference/assembly | 0.087 | 0.080 | 0.274 | -0.0691 | 0.2436 | 40.07 | 1.36 | QE(df = 20) = 20.28, p-val = 0.441 | QM(df = 1) = 1.20, p-val = 0.274 |
| Curriculum materials | -0.021 | 0.066 | 0.757 | -0.1505 | 0.1094 | 0 | 6.41 | QE(df = 20) = 21.37, p-val = 0.376 | QM(df = 1) = 0.10, p-val = 0.757 |
| Cooperative groupwork | 0.025 | 0.067 | 0.714 | -0.1070 | 0.1561 | 0 | 6.34 | QE(df = 20) = 21.35, p-val = 0.377 | QM(df = 1) = 0.13, p-val = 0.714 |
| Work with bully/victims | -0.069 | 0.060 | 0.252 | -0.1863 | 0.0488 | 65.23 | 0.79 | QE(df = 20) = 20.16, p-val = 0.448 | QM(df = 1) = 1.31, p-val = 0.252 |
| Work with peers | 0.087 | 0.036 | 0.016 | 0.0164 | 0.1578 | 100 | 0 | QE(df = 20) = 15.64, p-val = 0.739 | QM(df = 1) = 5.83, p-val = 0.016 |
| Information for parents/teachers | 0.006 | 0.063 | 0.925 | -0.1180 | 0.1298 | 0 | 6.82 | QE(df = 20) = 21.46, p-val = 0.370 | QM(df = 1) = 0.01, p-val = 0.925 |
| Improved playground supervision | 0.045 | 0.039 | 0.253 | -0.0320 | 0.1214 | 68.34 | 0.68 | QE(df = 20) = 20.14, p-val = 0.449 | QM(df = 1) = 1.31, p-val = 0.253 |
| Non-punitive methods | 0.009 | 0.046 | 0.852 | -0.0808 | 0.0979 | 0 | 6.61 | QE(df = 20) = 21.42, p-val = 0.373 | QM(df = 1) = 0.04, p-val = 0.852 |
| Teacher training | 0.036 | 0.050 | 0.470 | -0.0617 | 0.1339 | 0 | 4.25 | QE(df = 20) = 20.89, p-val = 0.404 | QM(df = 1) = 0.52, p-val = 0.470 |
| Parent training/methods | 0.031 | 0.044 | 0.476 | -0.0548 | 0.1175 | 0 | 4.22 | QE(df = 20) = 20.88, p-val = 0.404 | QM(df = 1) = 0.51, p-val = 0.476 |
| Videos and VR computer games | 0.041 | 0.036 | 0.263 | -0.0306 | 0.1119 | 56.11 | 0.92 | QE(df = 20) = 20.18, p-val = 0.446 | QM(df = 1) = 1.25, p-val = 0.263 |
| Internalizing symptoms | -0.026 | 0.042 | 0.530 | -0.1085 | 0.0558 | 0 | 4.80 | QE(df = 20) = 21.01, p-val = 0.397 | QM(df = 1) = 0.40, p-val = 0.529 |
| Social skills training | -0.015 | 0.058 | 0.795 | -0.1292 | 0.0989 | 0 | 6.52 | QE(df = 20) = 21.40, p-val = 0.374 | QM(df = 1) = 0.07, p-val = 0.795 |
| Empathy training | 0.001 | 0.046 | 0.988 | -0.0887 | 0.0902 | 0 | 6.84 | QE(df = 20) = 21.47, p-val = 0.370 | QM(df = 1) = 0.00, p-val = 0.988 |
| CBT elements | -0.079 | 0.036 | 0.0290 | -0.1490 | -0.0081 | 100 | 0 | QE(df = 20) = 16.70, p-val = 0.673 | QM(df = 1) = 4.78, p-val = 0.029 |

**Note**: Disciplinary methods and school tribunals were not included in the analysis as no studies included these components.

**Figure S1. Risk of Bias Assessment Plot**

**
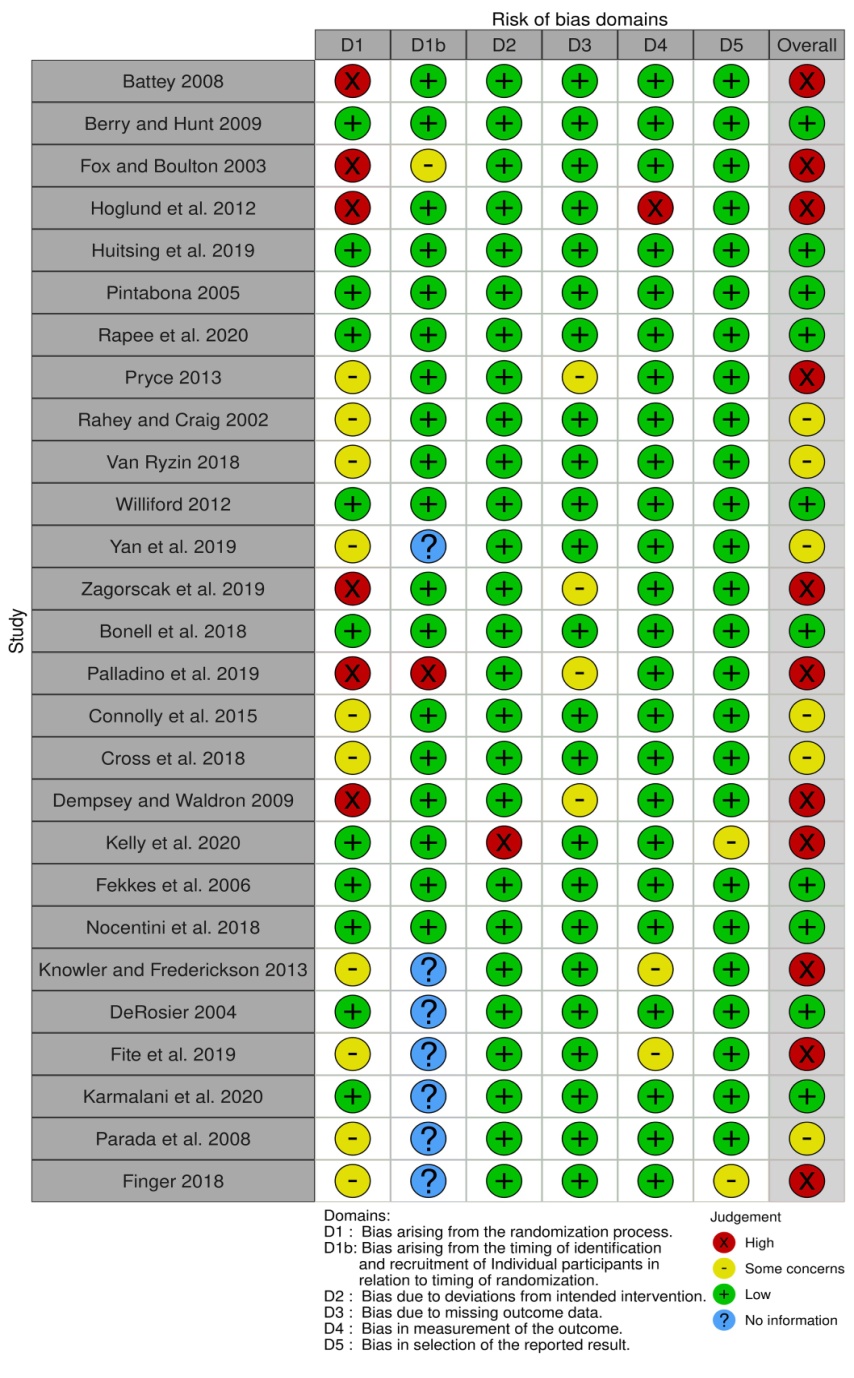
**

**Risk of bias assessment for all included studies**

**Citation**: McGuinness, LA, Higgins, JPT. Risk-of-bias VISualization (robvis): An R package and Shiny web app for visualizing risk-of-bias assessments. Res Syn Meth. 2020; 1- 7. https://doi.org/10.1002/jrsm.1411

**Figure S2. Duval and Tweedie trim-and-fill Funnel Plot**


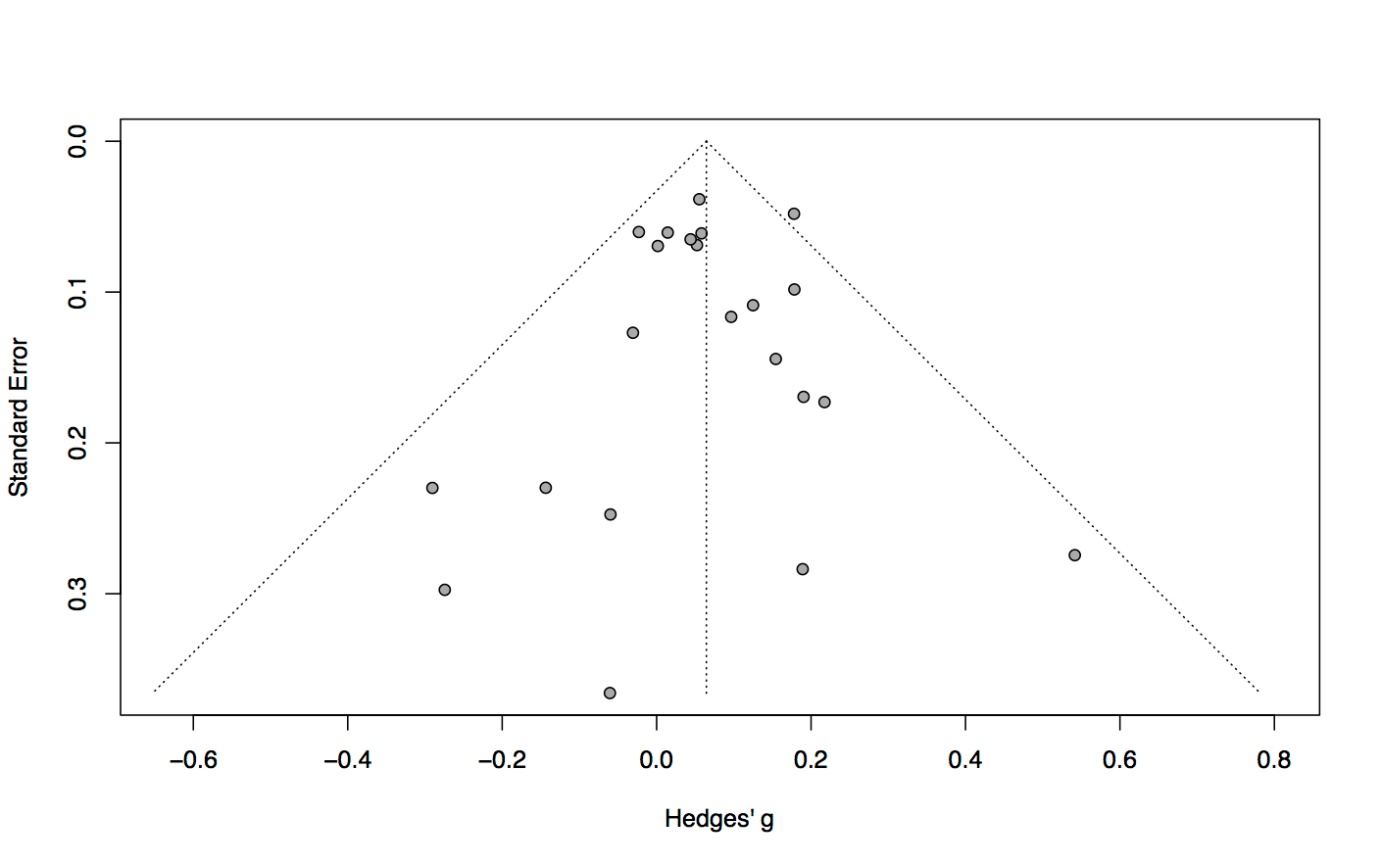


**Figure S3. Mediation Model for Bullying Victimisation**


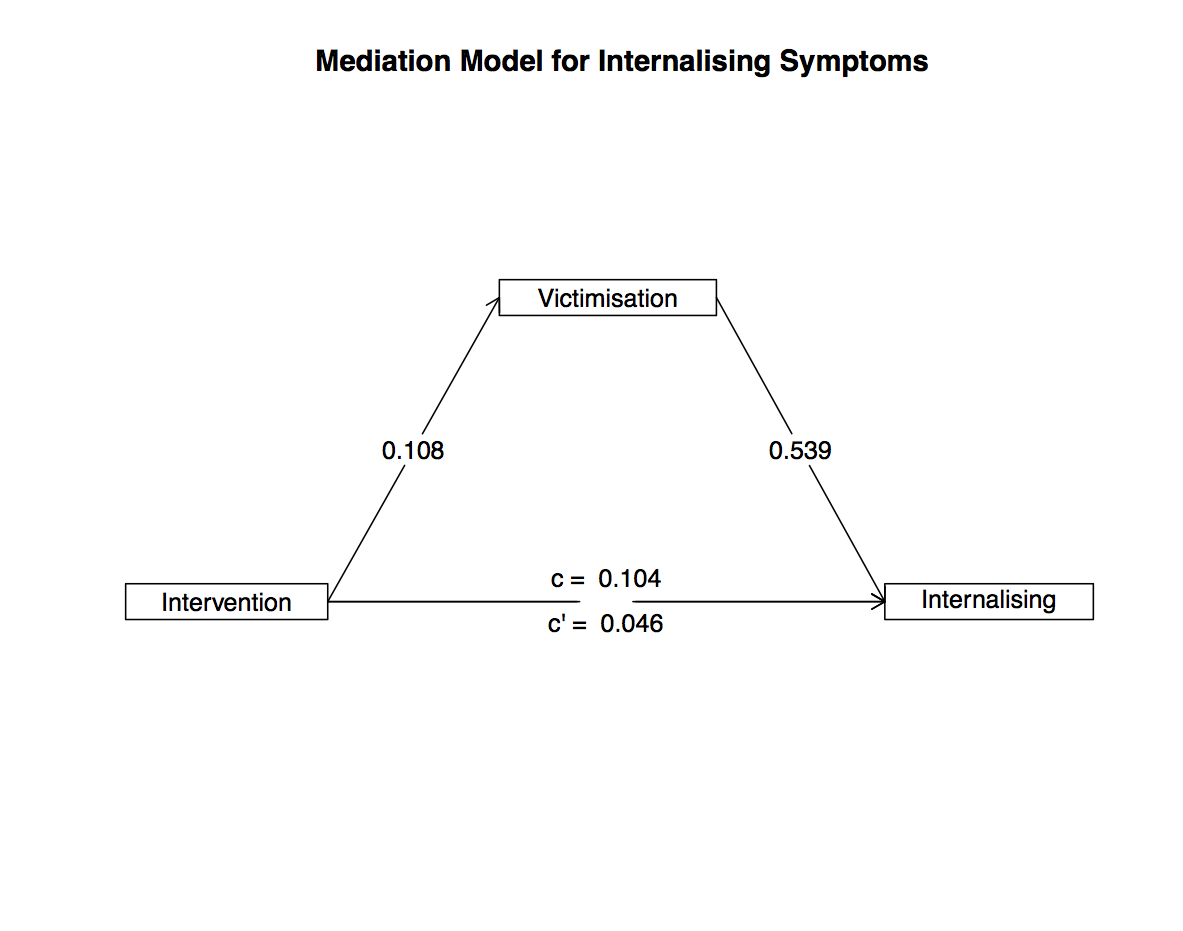


This figure shows that changes in pre-post bullying victimization did not mediate intervention effects on overall internalizing symptoms.

**Figure S4. Mediation Model for Bullying Perpetration**


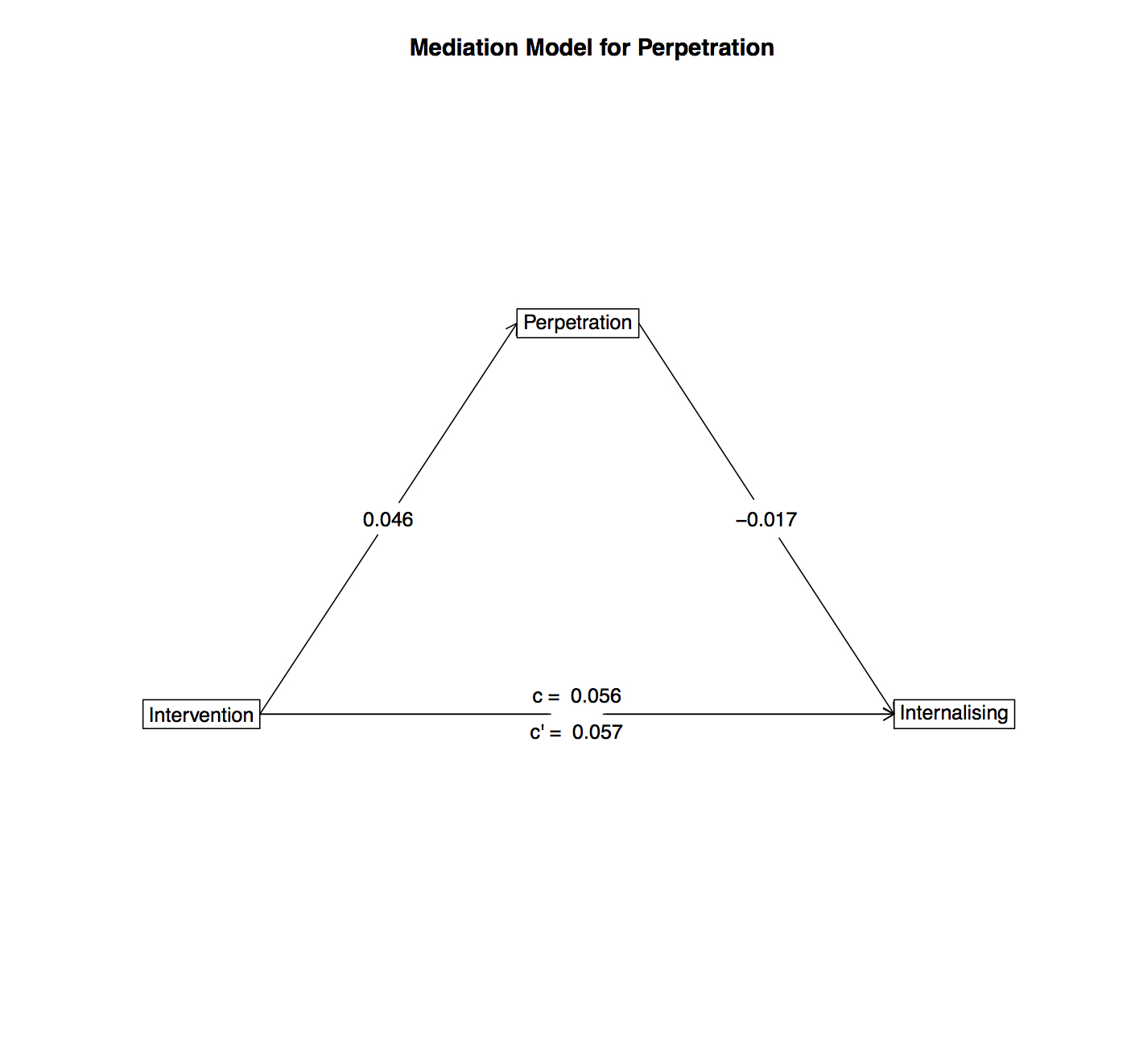


This figure shows that changes in pre-post bullying perpetration did not mediate intervention effects on overall internalizing symptoms.
